# Predictive Performance of the Cardiovascular Event Risk Test 2 Risk Score in Hemodialysis Patients with ESKD

**DOI:** 10.2215/CJN.0000000831

**Published:** 2025-11-12

**Authors:** Angelika Witoslawska, Christine Drechsler, Kevin Duarte, Nicolas Girerd, Faiez Zannad, Patrick Rossignol, Marcus E. Kleber, Hubert Scharnagl, Christoph Wanner, Winfried März, Reijo Laaksonen

**Affiliations:** 1 Finnish Cardiovascular Research Center, University of Tampere, Tampere, Finland; 2 Department of Clinical Research and Epidemiology, University Hospital of Würzburg, Würzburg, Germany; 3 Interdisciplinary Center for Palliative Medicine, University Hospital, Würzburg, Germany; 4 Centre d'Investigation Clinique Plurithémathique Pierre Drouin and Département de Cardiologie, Institut Lorrain du Coeur et des Vaisseaux, CHU de Nancy, Nancy, France; 5 Department of Medicine I (Cardiology, Hemostaseology, Medical Intensive Care), Medical Faculty Mannheim, University of Heidelberg, Mannheim, Germany; 6 SYNLAB MVZ Humangenetik Mannheim, Mannheim, Germany; 7 Clinical Institute of Medical and Chemical Laboratory Diagnostics, Medical University of Graz, Graz, Austria; 8 Zora Biosciences Oy, Espoo Finland

**Keywords:** cardiovascular disease, ESKD

## Abstract

**Key Points:**

- The Cardiovascular Event Risk Test 2 (CERT2) risk score effectively stratified patients undergoing hemodialysis patients on the basis of their risk of major adverse cardiovascular (CV) event, CV death, and all-cause death.
- Patients in the highest CERT2 risk category had a two-fold higher risk of major adverse CV event and all-cause death compared with those in the lowest category.
- CERT2 risk score shows that novel lipid-based biomarker solution outperforms regular CV risk biomarkers and existing risk scores in dialysis.

**Background:**

ESKD is closely associated with cardiovascular disease (CVD) primarily because of shared underlying conditions, such as diabetes mellitus and hypertension. Increased levels of circulating ceramide lipids have been linked to major adverse events in CVD, as well as to ESKD. Our aim was to investigate the predictive value of the Cardiovascular Event Risk Test 2 (CERT2) risk score in patients with ESKD in identifying patients at an increased risk of major adverse cardiovascular events (MACEs).

**Methods:**

This study combines data from 2311 patients from the A Study to Evaluate the Use of Rosuvastatin in Subjects on Regular Hemodialysis: An Assessment of Survival and Cardiovascular Events (AURORA) trial and 1137 patients from the Die Deutsche Diabetes Dialyze Studie (4D) trial. Both studies enrolled patients with ESKD undergoing hemodialysis. The CERT2 risk score has been calculated on the basis of quartile stratification of predefined lipid ratios for all the participants. The primary outcome of interest was MACE with additional outcomes of cardiovascular death and all-cause death.

**Results:**

After adjusting for traditional CV factors and other variables, two-fold higher risk was reported for MACE for patients in the highest risk category (AURORA: hazard ratios [HRs], 1.98; 95% confidence interval [CI], 1.53 to 2.55; 4D: HR, 1.70; 95% CI, 1.23 to 2.34) in comparison with those in the lowest risk category. Similarly, the two-fold higher risk was also observed for all-cause death for patients in the highest risk category (AURORA: HR, 2.14; 95% CI, 1.72 to 2.66; 4D: HR, 1.78; 95% CI, 1.36 to 2.34). In both trials, the CERT2 risk score demonstrated superior predictive performance compared with traditional markers like LDL cholesterol. In the AURORA cohort, the CERT2 risk score outperformed Systematic Coronary Risk Evaluation 2 and atherosclerotic CVD risk estimators in predicting CV outcomes.

**Conclusions:**

The CERT2 risk score identifies patients with ESKD who are at a higher risk of MACE and all-cause death. These findings highlight the potential of the CERT2 risk score as a novel lipid-based biomarker for CV risk stratification in patients undergoing hemodialysis, offering improved prognostic performance over existing biomarkers and risk scores.

## Visual Abstract

**Figure:**
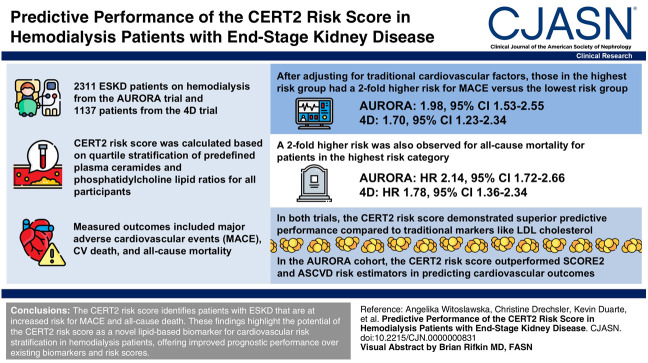

## Introduction

CKD affects nearly one billion people worldwide, and its prevalence is on the rise because of the aging population and increasing incidence rates of diabetes mellitus and hypertension.^1^ CKD can worsen over time and result in ESKD.

Kidney disease is closely associated with cardiovascular disease (CVD). This relationship is driven by shared underlying conditions, such as diabetes mellitus, hypertension, and atherosclerosis.^2^ CVD is not only highly prevalent in patients with ESKD but also represents the leading cause of increased risk of CV events and death.^3,4^ In fact, CVD is one of the main causes of morbidity and mortality in patients with ESKD.^5,6^ Moreover, the risk of cardiovascular (CV) events and mortality is even more pronounced in patients with ESKD and type 2 diabetes mellitus.^7^

Current treatment options for ESKD include KRT, either dialysis or organ transplantation, which are life-saving interventions.^8^ In 2010, over 2.5 million people were receiving some form of KRT and that number is projected to double by 2040.^9^ Patients undergoing dialysis face additional challenges, such as significantly increased risk of CVD complications and poor long-term outcomes.

Ceramides are complex sphingolipids that have been implicated in pathogenesis of metabolic diseases. They are composed of a sphingoid base linked to a fatty acid *via* an amide bond. Elevated levels of certain plasma ceramides, mainly C16:0, C18:0, and C24:1, have been strongly associated with major adverse outcomes in patients with CVD.^10^ Specifically, C16:0 denotes a ceramide with a 16-carbon saturated fatty acid (palmitic acid), C18:0 indicates an 18-carbon saturated fatty acid (stearic acid), and C24:1 represents a 24-carbon monounsaturated fatty acid (nervonic acid). Increased ceramide levels have also been linked to ESKD.^11^

Phosphatidylcholines (PCs) are a major class of glycerophospholipids integral to cell membrane integrity and lipid metabolism. Alterations in PC levels and their ratios with ceramides have been implicated in the pathogenesis of CVD, providing additional prognostic information beyond traditional lipid markers.

On the basis of those lipids, the CV Event Risk Test (CERT) has been developed to enhance CV risk prediction in patients with coronary artery disorder and acute coronary syndrome.^12^ The first-generation score, CV Event Risk Test 1 (CERT1), incorporates three ceramide ratios predictive of CV events. CV Event Risk Test 2 (CERT2), an updated version, includes additional PC species, which improve its discriminative power.^12,13^ Specifically, CERT2 comprises one ceramide-to-ceramide ratio, two ceramide-to-PC ratios, and a single PC species, reflecting a more comprehensive lipidomic profile.

Although the utility of CERT scores has been demonstrated in the coronary artery disorder populations, to our knowledge, their application to patients with ESKD undergoing hemodialysis has not been studied. Given the shared pathophysiologic mechanisms between CVD and ESKD, evaluating the applicability of CERT scores in the ESKD population may offer valuable prognostic insights. Therefore, using the data obtained from the A Study to Evaluate the Use of Rosuvastatin in Subjects on Regular Hemodialysis: An Assessment of Survival and Cardiovascular Events (AURORA) and Die Deutsche Diabetes Dialyze Studie (4D) trials, we have aimed to examine the relationship between distinct plasma ceramide and phospholipid species and the risk of major CV events in patients with ESKD undergoing hemodialysis.

## Methods

### Study Populations

The AURORA study design and main results have been reported.^14^ The trial randomized 2776 patients to either rosuvastatin or placebo, between age 50 and 80 years, with ESKD who were undergoing hemodialysis or hemofiltration for at least 3 months. The median follow-up was 3.2 years. This study includes 2311 patients with baseline samples available for the CERT risk score analyses, 1147 in the placebo group and 1164 in the rosuvastatin group (Supplemental Figure 1A).

The 4D study design and main results have been previously reported.^15^ The trial randomized 1255 patients with type 2 diabetes mellitus to either atorvastatin or placebo who were receiving maintenance hemodialysis. The median follow-up was 4 years. This study includes 1137 patients with baseline samples available for the CERT risk score analyses, 584 in the placebo group and 553 in the atorvastatin group (Supplemental Figure 1B).

Both studies were conducted in accordance with the Declaration of Helsinki, Good Clinical Practice guidelines, and the International Conference on Harmonization of Technical Requirements regulations. All participating centers had obtained approval from ethics committees or Institutional Review Boards. Written informed consent was obtained from all patients.

The baseline characteristics of the study populations can be found in Table 1.

**Table 1:** Baseline characteristics of the study cohorts

Characteristic	AURORA (*N*=2311)	4D (*N*=1137)
Age (yr)	64 (56–72)	66 (61–72)
Female sex	877 (38)	512 (45)
BMI (kg/m^2^)	24.5 (22.0–27.7) (*n*=2282)	26.8 (24.4–30.4) (*n*=1122)
Systolic BP (mm Hg)	137 (120–150) (*n*=2309)	142 (130–160) (*n*=1136)
Diastolic BP (mm Hg)	78 (68–83) (*n*=2308)	80 (70–80) (*n*=1135)
Systemic hypertension	1180/2309 (51)	752/1136 (66)
Current smoker	357 (15)	103 (9)
Total cholesterol (mmol/L)	4.40 (3.78–5.18) (*n*=2298)	5.53 (4.86–6.31)
LDL cholesterol (mmol/L)	2.49 (1.95–3.10) (*n*=2298)	3.22 (2.70–3.82) (*n*=1060)
HDL cholesterol (mmol/L)	1.09 (0.91–1.35) (*n*=2298)	0.88 (0.70–1.11)
Triglycerides (mmol/L)	1.46 (1.05–2.09) (*n*=2298)	2.50 (1.69–3.70)
hsCRP (mg/L)	5.1 (2.1–14.2) (*n*=2298)	5.1 (2.3–12.4) (*n*=1132)
Hemoglobin (g/dl)	11.7 (10.7–12.7) (*n*=2216)	10.9 (10.0–11.8) (*n*=1136)
Albumin (g/dl)	40 (37–42) (*n*=2299)	38 (36–40)
Calcium (mmol/L)	2.33 (2.20–2.48) (*n*=2300)	2.29 (2.18–2.40)
Phosphate (mmol/L)	1.74 (1.42–2.10) (*n*=2299)	1.91 (1.58–2.26)
Fasting glucose (mmol/L)	5.1 (4.6–5.8) (*n*=2300)	8.0 (6.2–10.5) (*n*=1043)
Duration of treatment with hemodialysis (yr)	2.4 (1.0–4.5) (*n*=2310)	0.5 (0.2–1.0) (*n*=1136)
**Duration of dialysis sessions, h/wk**	*n*=2310	*n*=1136
<12	506 (22)	188 (17)
12	1410 (61)	552 (49)
>12	394 (17)	396 (35)
**Current dialysis treatment**		*n*=1130
Hemodialysis	2140 (93)	1053 (93)
HDF	171 (7)	77 (7)
**Filters/membranes**		
High flux	979 (42)	N/A
Low flux	1332 (58)	N/A
**Type of access**	*n*=2296	*n*=1136
Arteriovenous fistula	1837 (80)	955 (84)
Central dialysis catheter	272 (12)	77 (7)
Graft	187 (8)	104 (9)
**Medical history**		
Diabetes mellitus	592 (26)	1137 (100)
History of hypertension	1792 (78)	1014 (89)
CVD	908 (39)	N/A
Coronary heart disease	1173 (51)	243 (21)
Myocardial infarction	234 (10)	203 (18)
Coronary revascularization	133 (6)	145 (13)
Peripheral vascular disease	339 (15)	508 (45)
**Drug therapy**		
Study drug		
*Placebo*	1147 (50)	584 (51)
*Statin*	1164 (50)	553 (49)
ACEI/ARB	841 (36)	667 (59)
Calcium-channel blocker	818 (35)	466 (41)
*β*-blocker	853 (37)	431 (38)
Diuretic	684 (30)	909 (80)
Platelet inhibitor	978 (42)	590 (52)
Vitamin D	1076 (47)	591 (52)
Calcium substitution	1723 (75)	N/A
Sevelamer	668 (29)	N/A
Erythropoietin	2040 (88)	N/A

Values are presented as median (Q1–Q3) or frequency (%). 4D, Die Deutsche Diabetes Dialyze Studie; ACEI, angiotensin-converting-enzyme inhibitor; ARB, angiotensin II receptor blocker; AURORA, A Study to Evaluate the Use of Rosuvastatin in Subjects on Regular Hemodialysis: An Assessment of Survival and Cardiovascular Events; BMI, body mass index; CVD, cardiovascular disease; HDF, hemodiafiltration; hsCRP, high-sensitivity C-reactive protein; N/A, not available.

### Sample Collection and Processing

In both the AURORA and 4D trials, blood samples were collected before dialysis sessions to ensure consistency and minimize dialysis-related variability in lipid levels. Blood collection occurred either in the morning following an overnight fast or in the afternoon at least 6 hours after a standardized carbohydrate-containing breakfast. Alcohol intake and smoking were prohibited within 24 hours before blood collection.

In both studies, plasma samples were stored at −80°C until analysis.

### End Point Assessment

The primary end point for this analysis was major adverse CV events (MACEs), *i.e*., a three-point MACE defined as a composite end point of nonfatal myocardial infarction, nonfatal stroke, or CV death. The end point assessment investigated the association between three-point MACE and plasma lipid variables while using the prognostic value of the CERT risk score. Assessment also included additional outcomes of interest, specifically CV death and all-cause death.

### Prognostic Scores

This analysis focuses on the CERT2 risk score, an improved successor to CERT1 with greater prognostic value (Supplemental Table 1). The CERT2 risk score combines distinct plasma Cer and PC lipid ratios along with a single PC:

1. Cer(d18:1/24:1)/Cer(d18:1/24:0).
2. Cer(d18:1/16:0)/PC(16:0/22:5).
3. Cer(d18:1/18:0)/PC(14:0/22:6).
4. PC(16:0/16:0).

To calculate the CERT2 score, patients were assigned a score between 0 and 3 for quartiles 1–4. The four components were added together, and then the score, ranging from 0 to 12, was stratified into four risk categories from low to high risk. Quartile-based scoring for CERT2 was performed separately within each cohort (AURORA and 4D) to account for potential differences in lipid distributions and cohort characteristics. The detailed calculation of the CERT2 risk score has been previously described.^13^

### Systematic Coronary Risk Evaluation 2 and Atherosclerotic CVD

Systematic Coronary Risk Evaluation 2 (SCORE2) and atherosclerotic CVD (ASCVD) risk scores were calculated and compared with CERT2 in predictive modeling for AURORA cohort. SCORE2 estimates 10-year CV risk using age, sex, smoking status, systolic BP, and total and HDL cholesterol. It stratifies patients into four regional risk categories defined by the 2021 European Society of Cardiology guidelines.^16^ Because country of residence data were unavailable and the study population comprises patients with ESKD, who are universally considered at high CV risk, SCORE2 results were restricted to the very-high-risk-region classification. The ASCVD risks score, developed by the American College of Cardiology and the American Heart Association, similarly estimates 10-year CV risk consisting of age, sex, race, total and HDL cholesterol, BP, smoking, and diabetes status.^17^ A detailed description of both scores in the AURORA population is provided in Supplemental Table 2.

### Quantification of Ceramides and PCs

The plasma levels of Cer(d18:1/16:0), Cer(d18:1/18:0), Cer(d18:1/24:0), Cer(d18:1/24:1), PC(14:0/22:6), PC(16:0/16:0), and PC(16:0/22:5) were quantified on a 5500 quadrupole linear ion trap mass spectrometer equipped with ultra-high-performance liquid chromatography system. PCs were analyzed from the same lipid extract as the ceramides.

### Statistical Analyses

Baseline characteristics, CV risk factors, and lipid variables (*i.e*., lipids, lipid ratios, and CERT2 risk score) were compared between the AURORA and 4D cohorts using the nonparametric Wilcoxon test for continuous variables and the Fisher exact test for categorical variables.

Association between lipid variables and study end points (three-point MACE, CV death, or all-cause death) was performed using time-to-event analyses using Cox regression model. The following three progressive Cox regression models were used: model 1: unadjusted; model 2: adjusted for age and sex; and model 3: adjusted for age, sex, body mass index (BMI), systemic hypertension, type 2 diabetes mellitus, smoking, log high-sensitivity C-reactive protein (hsCRP) and treatment group. Hazard ratios (HRs) are presented with their 95% confidence intervals (CIs). *P* values were adjusted for multiple comparisons using Benjamini–Hochberg method to control the false discovery rate across all 90 tests (10 biomarkers×3 outcomes×3 models).

MACE-free survival rates, CV survival rates, and survival rates according to lipid groups (quartiles for lipids and lipid ratios, risk groups for CERT1 and CERT2 scores) were illustrated using the Kaplan–Meier analyses. Difference between survival curves was analyzed using the log-rank test.

To evaluate the associations between the lipid variables and the risk factors of CVD, Spearman rank correlation coefficients was used. To evaluate predictive performance of the CERT2 risk score, Harrell concordance index (c-index) was calculated for univariable models and for models incorporating CERT2 alongside LDL cholesterol and comprehensive clinical variables.

Continuous variables were expressed as median (interquartile range) and categorical variables as frequency (%). Missing data were not imputed; analyses were performed on available data only. All statistical analyses were conducted using R software (R Foundation for Statistical Computing). The significance level was set at *P* value < 0.05.

## Results

### Overview of the AURORA and 4D Cohorts

Although both AURORA and 4D enrolled patients with ESKD undergoing maintenance hemodialysis, there were notable differences between the two populations (Table 1). Most significantly, the 4D trial enrolled exclusively patients with type 2 diabetes mellitus, whereas only 26% of AURORA participants had type 2 diabetes mellitus.

Patients in the 4D cohort were slightly older (median age 66 versus 64 years), more frequently female (45% versus 38%), and had a higher BMI (26.8 versus 24.5 kg/m^2^). They also exhibited worse metabolic and CV profiles. CV risk factors, such as systemic hypertension (66% versus 51%) and a history of hypertension (89% versus 78%), were more prevalent in 4D cohort. However, coronary heart disease was more common in AURORA cohort (51% versus 21%), whereas peripheral vascular disease was more prevalent in 4D (45% versus 15%).

The dialysis duration also differed substantially. AURORA patients had a median of 2.4 years on hemodialysis versus just 0.5 years in 4D. A greater proportion of 4D participants received longer dialysis sessions. In both trials, most patients received conventional hemodialysis with arteriovenous fistula as the preferred vascular access.

### Association of Individual Lipid Species with All-Cause Death and CV End Points

Various plasma ceramide and PC species, including their distinct ratios, have been analyzed to determine their potential prognostic value. Significant associations have been observed across a wide range of lipids, lipid ratios, and all three end points, before and after adjusting for clinical covariates and for multiple comparisons (Supplemental Tables 3 and 4). Among the individual lipid species, significant associations for all three end points were reported for PC(14:0/22:6), Cer(d18:1/14:0), PC(16:0/22:5), and Cer(d18:1/16_0) in both the AURORA and 4D cohorts. Two Cer and PC lipid ratios, specifically Cer(d18:1/16:0)/PC(16:0)/(22:5) and Cer(d18:1/18:0)/PC(14:0/22:6), appear to be the driving force in the association of the CERT2 risk score with CV end points in both AURORA and 4D trials. Following an adjustment for clinical variables (BMI, systemic hypertension, type 2 diabetes mellitus, smoking, log hsCRP, treatment group, model 3), both ratios have significant associations with the three-point MACE, CVD death, and all-cause mortality. Further adjustment for multiple corrections has preserved significant associations for all but CV death in 4D cohort.

### CERT2 Risk Score and Study End Points

**Figure 1:**
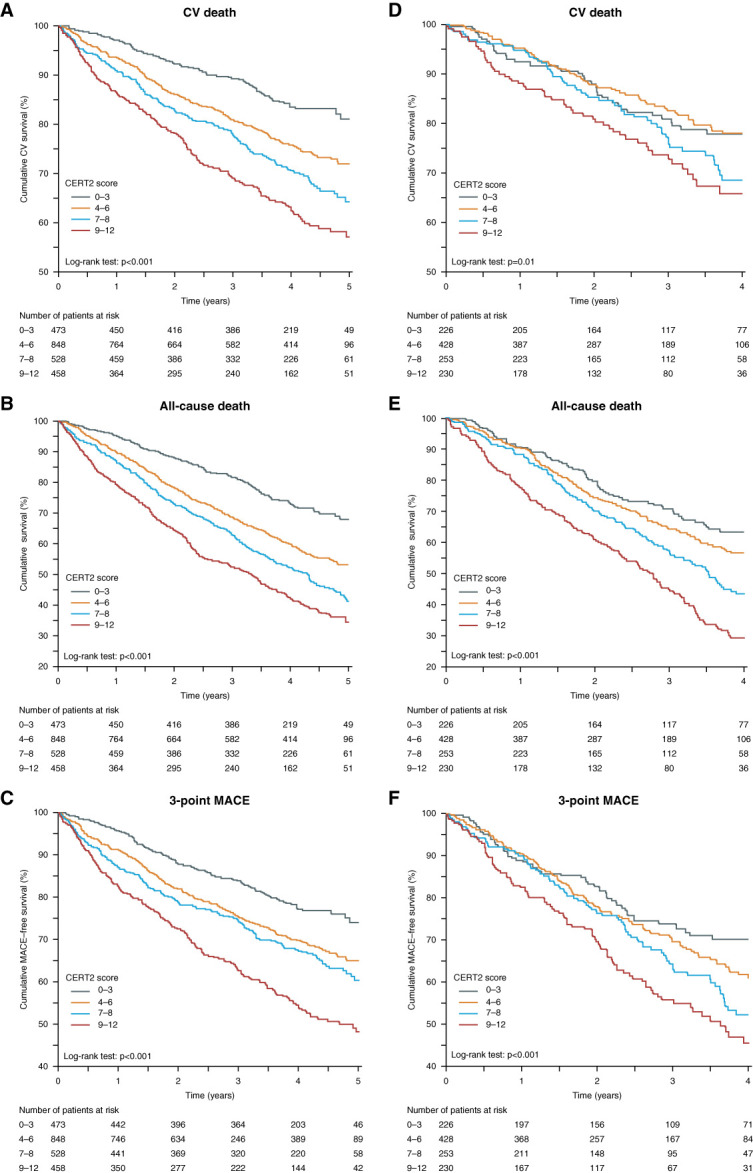
**A higher CERT2 risk score is associated with an increased risk of CV death, all-cause death, and MACEs.** Kaplan–Meier curves for CERT2 in (A–C) AURORA and (D–F) 4D cohorts, showing cumulative incidence of CV death, all-cause death and MACE. The number of patients at risk is shown below each plot. 4D, Die Deutsche Diabetes Dialyze Studie; AURORA, A Study to Evaluate the Use of Rosuvastatin in Subjects on Regular Hemodialysis: An Assessment of Survival and Cardiovascular Events; CERT2, Cardiovascular Event Risk Test 2; CV, cardiovascular; MACE, major adverse cardiovascular event.

The study evaluated the associations between the CERT2 risk score, a three-point MACE, CV death, and all-cause death. In the AURORA trial, there was a 2.5-fold higher risk of the three-point MACE (Figure 1 and Table 2) in the highest risk group compared with the lower risk group before adjusting for clinical covariates (unadjusted HR, 2.47; 95% CI, 1.94 to 3.13). A 2.8-fold higher risk of CV death (unadjusted HR, 2.77; 95% CI, 2.11 to 3.65) and 2.9-fold higher risk of all-cause death (unadjusted HR, 2.86; 95% CI, 2.33 to 3.51) were also reported in the highest risk group patients before adjustment for clinical covariates. Similar trend was observed in the 4D trial, and the risk of all three end points were significantly higher with greater CERT2 score (Figure 1 and Table 3).

**Table 2:** Cardiovascular Event Risk Test 2 risk score and its association with cardiovascular death, all-cause death and three-point major adverse cardiovascular event in the A Study to Evaluate the Use of Rosuvastatin in Subjects on Regular Hemodialysis: An Assessment of Survival and Cardiovascular Events trial

AURORA	No. of Events	No. of Person-years	Event Rate (per 1000 Person Years)	Model 1 (*n*=2307)		Model 2 (*n*=2307)		Model 3 (*n*=2265)	
				HR (95% CI)	*P* Value	HR (95% CI)	*P* Value	HR (95% CI)	*P* Value
**CV death**									
CERT2 score									
*0–3*	76	1774	43	Reference	—	Reference	—	Reference	—
*4–6*	202	2943	69	1.60 (1.23 to 2.09)	<0.001^a^	1.53 (1.17 to 1.99)	0.002^a^	1.51 (1.15 to 1.98)	0.003^a^
*7–8*	151	1722	88	2.05 (1.55 to 2.70)	<0.001^a^	1.91 (1.45 to 2.52)	<0.001^a^	1.79 (1.35 to 2.39)	<0.001^a^
*9–12*	158	1327	119	2.77 (2.11 to 3.65)	<0.001^a^	2.56 (1.94 to 3.37)	<0.001^a^	2.23 (1.67 to 2.99)	<0.001^a^
**All-cause death**									
CERT2 score									
*0–3*	136	1774	77	Reference	—	Reference	—	Reference	—
*4–6*	373	2943	127	1.66 (1.36 to 2.02)	<0.001^a^	1.58 (1.30 to 1.92)	<0.001^a^	1.51 (1.24 to 1.85)	<0.001^a^
*7–8*	283	1722	164	2.15 (1.76 to 2.64)	<0.001^a^	2.00 (1.63 to 2.46)	<0.001^a^	1.78 (1.44 to 2.20)	<0.001^a^
*9–12*	288	1327	217	2.86 (2.33 to 3.51)	<0.001^a^	2.62 (2.14 to 3.22)	<0.001^a^	2.14 (1.72 to 2.66)	<0.001^a^
**Three-point MACE**									
CERT2 score									
*0–3*	105	1701	62	Reference	—	Reference	—	Reference	—
*4–6*	254	2816	90	1.46 (1.16 to 1.83)	0.001^a^	1.40 (1.12 to 1.76)	0.004^a^	1.36 (1.08 to 1.72)	0.01^a^
*7–8*	170	1670	102	1.65 (1.29 to 2.10)	<0.001^a^	1.55 (1.21 to 1.98)	<0.001^a^	1.41 (1.10 to 1.82)	0.007^a^
*9–12*	193	1252	154	2.47 (1.94 to 3.13)	<0.001^a^	2.31 (1.81 to 2.93)	<0.001^a^	1.98 (1.53 to 2.55)	<0.001^a^

Model 1: unadjusted; model 2: adjusted for age, sex; model 3: adjusted for age, sex, body mass index, systemic hypertension, type 2 diabetes mellitus, smoking, log high-sensitivity C-reactive protein, treatment group (rosuvastatin versus placebo). AURORA, A Study to Evaluate the Use of Rosuvastatin in Subjects on Regular Hemodialysis: An Assessment of Survival and Cardiovascular Events; CERT2, Cardiovascular Event Risk Test 2; CI; confidence interval; CV, cardiovascular; HR, hazard ratio; MACE, major adverse cardiovascular event.

a *P*-value < 0.05.

**Table 3:** Cardiovascular Event Risk Test 2 risk score and its association with cardiovascular death, all-cause death, and three-point major adverse cardiovascular event in the Die Deutsche Diabetes Dialyze Studie trial

4D	No. of Events	No. of Person-Years	Event Rate (per 1000 Person Years)	Model 1 (*n*=1137)		Model 2 (*n*=1137)		Model 3 (*n*=1117)	
				HR (95% CI)	*P* Value	HR (95% CI)	*P* Value	HR (95% CI)	*P* Value
**CV death**									
CERT2 score									
*0–3*	49	701	70	Reference	—	Reference	—	Reference	—
*4–6*	80	1217	66	0.95 (0.66 to 1.35)	0.77	0.91 (0.63 to 1.30)	0.59	0.88 (0.61 to 1.25)	0.47
*7–8*	61	706	86	1.25 (0.86 to 1.82)	0.24	1.18 (0.81 to 1.72)	0.40	1.08 (0.73 to 1.59)	0.70
*9–12*	60	548	110	1.59 (1.09 to 2.33)	0.02^a^	1.54 (1.06 to 2.26)	0.02^a^	1.25 (0.84 to 1.86)	0.26
**All-cause death**									
CERT2 score									
*0–3*	89	701	127	Reference	—	Reference	—	Reference	—
*4–6*	181	1217	149	1.20 (0.93 to 1.54)	0.16	1.13 (0.88 to 1.46)	0.35	1.07 (0.83 to 1.38)	0.61
*7–8*	141	706	200	1.61 (1.23 to 2.10)	<0.001^a^	1.49 (1.14 to 1.95)	0.003^a^	1.31 (1.00 to 1.72)	0.05
*9–12*	157	548	287	2.36 (1.81 to 3.06)	<0.001^a^	2.27 (1.75 to 2.95)	<0.001^a^	1.78 (1.36 to 2.34)	<0.001^a^
**Three-point MACE**									
CERT2 score									
*0–3*	67	665	101	Reference	—	Reference	—	Reference	—
*4–6*	138	1114	124	1.25 (0.93 to 1.67)	0.14	1.21 (0.90 to 1.62)	0.20	1.18 (0.88 to 1.58)	0.28
*7–8*	97	644	151	1.52 (1.11 to 2.07)	0.009^a^	1.47 (1.07 to 2.01)	0.02^a^	1.37 (0.99 to 1.89)	0.05
*9–12*	98	504	195	1.98 (1.45 to 2.70)	<0.001^a^	1.93 (1.41 to 2.64)	<0.001^a^	1.70 (1.23 to 2.34)	0.001^a^

Model 1: unadjusted; model 2: adjusted for age, sex; model 3: adjusted for age, sex, body mass index, systemic hypertension, type 2 diabetes mellitus, smoking, log high-sensitivity C-reactive protein, treatment group (atorvastatin versus placebo). 4D, Die Deutsche Diabetes Dialyze Studie; CERT2, Cardiovascular Event Risk Test 2; CI; confidence interval; CV, cardiovascular; HR, hazard ratio; MACE, major adverse cardiovascular event.

a *P*-value < 0.05.

After adjusting for age and sex (model 2), the three-point MACE, CV death, and all-cause death remained significantly associated with increasing CERT2 score in both AURORA and 4D trials. After further adjustment for clinical covariables (BMI, systemic hypertension, type 2 diabetes mellitus, smoking, log hsCRP, and treatment group, model 3), a significant association for three-point MACE and the highest CERT2 score compared with the lowest risk score remained (AURORA: HR, 1.98; 95% CI, 1.53 to 2.55; 4D: HR, 1.70; 95% CI, 1.23 to 2.34). The significant association has also remained for all-cause death. The significance association between CV death and the CERT2 risk score has continued for the AURORA trial but was lost for the 4D trial.

### Predictive Performance of the CERT2 Score in the Cohorts

In both trials, the CERT2 risk score demonstrated superior predictive performance compared with traditional markers like LDL cholesterol. In the AURORA trial, the CERT2 score demonstrated modest yet statistically significant predictive capabilities for CV death, all-cause death, and MACE, with c-indices 0.593, 0.595, and 0.581, respectively (Supplemental Table 5). Compared with LDL cholesterol, CERT2 outperformed LDL in predicting these end points. Incorporating CERT2 into models including LDL cholesterol or comprehensive clinical variables further enhanced predictive accuracy across all end points. Similar results were seen in the 4D cohort (Supplemental Table 6), with CERT2 significantly outperforming LDL cholesterol in predicting all-cause death and MACE and modestly improving models that included LDL cholesterol and clinical variables.

To further contextualize predictive performance, the CERT2 risk score was compared with established risk scores, ASCVD and SCORE2, in the AURORA cohort. Associations between the scores and study end points were evaluated (Supplemental Table 7). ASCVD showed a 2.1-fold higher risk of three-point MACE (unadjusted HR, 2.10; 95% CI, 1.64 to 2.69), 1.9-fold for CV-death (unadjusted HR, 1.94; 95% CI, 1.49 to 2.54), and two-fold for all-cause death (unadjusted HR, 2.06; 95% CI, 1.69 to 2.51) in the highest risk group. SCORE2 showed similar associations. All HRs were consistently lower than those observed for CERT2.

The association between the CERT2 risk score and study end points was further assessed in models adjusted for either ASCVD or SCORE2 (Table 4). In all models, CERT2 remained independently and significantly associated with each end point. Moreover, in terms of predictive accuracy, the CERT2 risk score demonstrated a higher univariable c-index than both SCORE2 and ASCVD. Incorporating CERT2 into these established risk models further enhanced predictive performance, with all Δc-index (*i.e*., changes in concordance index) values exceeding 0.035 (Table 5).

**Table 4:** Association of Cardiovascular Event Risk Test 2 score with outcomes in A Study to Evaluate the Use of Rosuvastatin in Subjects on Regular Hemodialysis: An Assessment of Survival and Cardiovascular Events trial (univariable and adjusted separately for each cardiovascular risk score)

AURORA	No. of Events	No. of Person-Years	Event Rate (per 1000 Person Years)	Unadjusted (*n*=2292)		Adjusted for SCORE2-VHRR (*n*=2292)		Adjusted for ASCVD Score (*n*=2292)	
				HR (95% CI)	*P* Value	HR (95% CI)	*P* Value	HR (95% CI)	*P* Value
**CV death**									
CERT2 score									
*0–3*	75	1762	43	Reference	—	Reference	—	Reference	—
*4–6*	201	2922	69	1.62 (1.24 to 2.11)	<0.001^a^	1.58 (1.22 to 2.07)	<0.001^a^	1.59 (1.22 to 2.07)	<0.001^a^
*7–8*	151	1713	88	2.07 (1.57 to 2.74)	<0.001^a^	1.99 (1.51 to 2.63)	<0.001^a^	1.99 (1.51 to 2.63)	<0.001^a^
*9–12*	157	1317	119	2.79 (2.12 to 3.68)	<0.001^a^	2.66 (2.02 to 3.50)	<0.001^a^	2.64 (2.00 to 3.48)	<0.001^a^
**Death**									
CERT2 score									
*0–3*	134	1762	76	Reference	—	Reference	—	Reference	—
*4–6*	370	2922	127	1.67 (1.37 to 2.04)	<0.001^a^	1.64 (1.34 to 2.00)	<0.001^a^	1.64 (1.35 to 2.00)	<0.001^a^
*7–8*	283	1713	165	2.19 (1.78 to 2.69)	<0.001^a^	2.10 (1.71 to 2.58)	<0.001^a^	2.10 (1.71 to 2.58)	<0.001^a^
*9–12*	285	1317	216	2.87 (2.34 to 3.53)	<0.001^a^	2.74 (2.23 to 3.36)	<0.001^a^	2.71 (2.21 to 3.33)	<0.001^a^
**MACE**									
CERT2 score									
*0–3*	104	1690	62	Reference	—	Reference	—	Reference	—
*4–6*	252	2798	90	1.46 (1.16 to 1.84)	0.001^a^	1.44 (1.14 to 1.81)	0.002^a^	1.44 (1.15 to 1.81)	0.002^a^
*7–8*	169	1660	102	1.65 (1.29 to 2.11)	<0.001^a^	1.59 (1.24 to 2.02)	<0.001^a^	1.59 (1.25 to 2.03)	<0.001^a^
*9–12*	191	1247	153	2.46 (1.94 to 3.12)	<0.001^a^	2.35 (1.85 to 2.98)	<0.001^a^	2.35 (1.85 to 2.98)	<0.001^a^

ASCVD, atherosclerotic cardiovascular disease; AURORA, A Study to Evaluate the Use of Rosuvastatin in Subjects on Regular Hemodialysis: An Assessment of Survival and Cardiovascular Events; CERT2, Cardiovascular Event Risk Test 2; CI, confidence interval; CV, cardiovascular; HR, hazard ratio; MACE, major adverse cardiovascular event; SCORE2, Systematic Coronary Risk Evaluation 2; VHRR, very high-risk region.

a *P*-value < 0.05.

**Table 5:** Predictive performance of cardiovascular risk scores (Systematic Coronary Risk Evaluation 2, atherosclerotic cardiovascular disease) compared with Cardiovascular Event Risk Test 2 score, and incremental prognostic value of Cardiovascular Event Risk Test 2 on top of cardiovascular risk scores

AURORA	End Point	C-Index (95% CI)		
		Model 1 (CV Risk Score)	Model 2 (CERT2 Score)	Model 3 (CV Risk Score+CERT2 Score)
CV risk score=SCORE2-VHRR (*n*=2292)	CV death	0.55 (0.54 to 0.56)	0.59 (0.57 to 0.62)	0.62 (0.60 to 0.64)
	All-cause death	0.55 (0.54 to 0.56)	0.60 (0.58 to 0.61)	0.62 (0.60 to 0.63)
	MACE	0.55 (0.54 to 0.56)	0.58 (0.56 to 0.60)	0.61 (0.59 to 0.63)
CV risk score=ASCVD score (*n*=2292)	CV death	0.57 (0.55 to 0.59)	0.59 (0.57 to 0.62)	0.62 (0.60 to 0.64)
	All-cause death	0.58 (0.56 to 0.59)	0.60 (0.58 to 0.61)	0.62 (0.60 to 0.64)
	MACE	0.57 (0.55 to 0.59)	0.58 (0.56 to 0.60)	0.61 (0.59 to 0.63)

AURORA	End Point	Comparison of Predictive Performance between CV Risk Score and CERT2 Score (Model 1 versus Model 2)		Incremental Prognostic Value of CERT2 Score on Top of CV Risk Score (Model 1 versus Model 3)	
		Δc-Index (95% CI)	*P* Value	Δc-Index (95% CI)	*P* Value
CV risk score=SCORE2-VHRR (*n*=2292)	CV death	0.05 (0.02 to 0.07)	<0.001^a^	0.07 (0.05 to 0.09)	<0.001^a^
	All-cause death	0.05 (0.03 to 0.07)	<0.001^a^	0.07 (0.06 to 0.08)	<0.001^a^
	MACE	0.03 (0.01 to 0.05)	0.01^a^	0.06 (0.04 to 0.08)	<0.001^a^
CV risk score=ASCVD score (*n*=2292)	CV death	0.03 (−0.01 to 0.06)	0.11	0.05 (0.03 to 0.07)	<0.001^a^
	All-cause death	0.02 (−0.00 to 0.04)	0.08	0.05 (0.03 to 0.06)	<0.001^a^
	MACE	0.01 (−0.01 to 0.04)	0.35	0.04 (0.02 to 0.06)	<0.001^a^

ASCVD, atherosclerotic cardiovascular disease; AURORA, A Study to Evaluate the Use of Rosuvastatin in Subjects on Regular Hemodialysis: An Assessment of Survival and Cardiovascular Events; CERT2, Cardiovascular Event Risk Test 2; Δc-Index, changes in concordance index; CI, confidence interval; CV, cardiovascular; MACE, major adverse cardiovascular event; SCORE2, Systematic Coronary Risk Evaluation 2; VHRR, very high-risk region.

a *P*-value < 0.05.

### Correlation between Lipid Variables and Traditional Risk Factors of CVD

**Figure 2:**
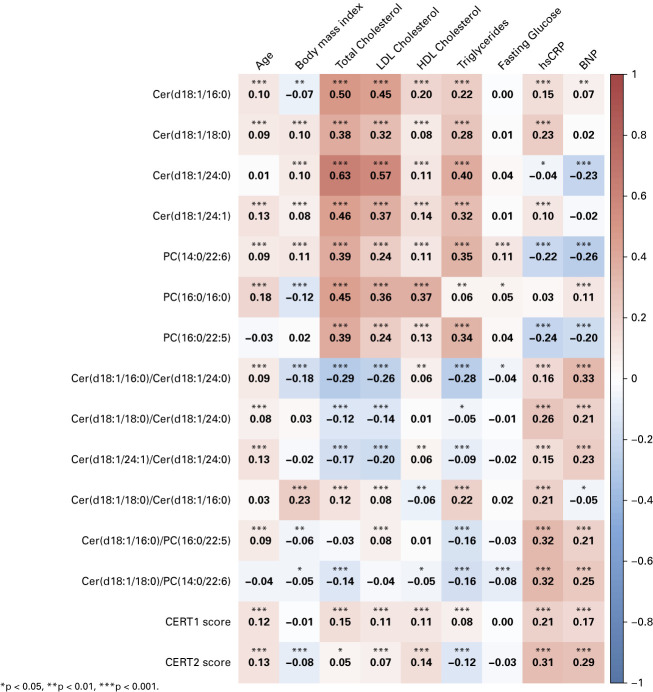
**Lipid variables show significant correlations with traditional CV risk factors.** Heatmap illustrating pairwise correlations between lipid variables that are components of the CERT risk scores and traditional CV risk factors in the AURORA cohort. BNP, brain natriuretic peptide; CERT, Cardiovascular Event Risk Test; hsCRP, high-sensitivity C-reactive protein.

In the AURORA trial, further analysis was performed to explore the relationship between specific lipid variables and traditional risk factors of CVD using Spearman correlation coefficients (Figure 2). The following risk factors were investigated: age, male sex, BMI, smoking status, systemic hypertension, type 2 diabetes mellitus, total cholesterol, LDL cholesterol, HDL cholesterol, triglycerides, fasting glucose, hsCRP, and brain natriuretic peptide (BNP). Single ceramide species showed weak-to-moderate correlation with the CV risk factors. The strongest correlations were observed between ceramides and standard lipid variables such as total cholesterol, LDL cholesterol, triglycerides, and HDL cholesterol. The CERT2 risk score showed weak correlations with CVD risk factors, and the strongest correlations were observed with hsCRP (Spearman *r*=0.31, *P* < 0.001) and BNP (Spearman *r*=0.29, *P* < 0.001), whereas with triglycerides, a negative correlation was reported.

## Discussion

This study has demonstrated that a plasma ceramide and phospholipid-based risk score (CERT2) allows the identification of patients with ESKD who are at a higher risk of MACE. The CERT2 risk score can also efficiently identify those individuals who are at a higher risk of all-cause death. Importantly, the CERT2 risk score outperformed traditional CV risk factors such as LDL cholesterol in predictive value, highlighting its potential utility in a population where conventional markers fall short.

Increased levels of circulating plasma ceramides have been linked to many cardiac and metabolic pathologies.^18^ Given their strong involvement in disease development and progression, plasma ceramides can be used as prognostic markers. In previous studies, certain ceramide species, mainly Cer(d18:1/16:0), Cer(d18:1/18:0), and Cer(d18:1/24:1), have been significantly associated with major CV events regardless of traditional ESKD risk factors.^19^ In patients with kidney disease, greater plasma ceramide levels of Cer(d18:1/16:0), Cer(d18:1/18:0), and Cer(d18:1/24:1) were shown to be significantly associated with a higher risk of major CV events.^11^

In our study of patients with ESKD, we observed that Cer(d18:1/16:0) was significantly associated with all three end points in the AURORA trial. In the 4D trial, Cer(d18:1/16:0) was significantly associated with all-cause mortality but not with CV death or MACE. By contrast, Cer(d18:1/18:0) and Cer(d18:1/24:1) did not show significant associations with any of the end points in either cohort. These findings suggest that Cer(d18:1/16:0) may be a robust biomarker for mortality risk in patients with ESKD, particularly those undergoing hemodialysis. The lack of association for Cer(d18:1/18:0) and Cer(d18:1/24:1) may be attributed to the unique pathophysiologic conditions present in patients with ESKD, such as altered lipid metabolism and systemic inflammation, which could affect ceramide profiles and their associations with clinical outcomes.

Traditional CVD risk factors have also been implicated in the development of CVD in patients with ESKD. Hypertension, insulin resistance, smoking, and dyslipidemia not only contribute to the development of CVD, but they also contribute to the progression of ESKD.^20^ For instance, hypertension is prevalent in patients with ESKD and contributes to left ventricular hypertrophy and heart failure.^21,22^ Previously, Framingham study has pointed out that traditional CVD risk factors do not accurately predict the CV events in patients with ESKD.^23^ In our analysis, the CERT2 risk score demonstrated only modest correlations with traditional risk factors. Specifically, CERT2 showed moderate association with hsCRP and BNP. Associations with age and HDL cholesterol were mild, whereas correlations with total and LDL cholesterol were minimal. An inverse association was observed for triglycerides and BMI. These findings suggest that CERT2 captures aspects of CV risk not reflected by traditional risk factors and may enhance risk stratification in this population.

In both AURORA and 4D trials, the CERT2 score has demonstrated superior predictive performance for CV and all-cause death compared with traditional markers like LDL cholesterol. In the AURORA trial, CERT2 consistently outperformed LDL cholesterol across all end points with statistically significant improvements in c-indices. Similar observation can be made for 4D. Moreover, integrating CERT2 into existing clinical models, including those accounting for LDL-cholesterol, yielded modest yet statistically significant improvements in risk prediction, particularly for all-cause death. This suggests that the CERT2 score provides additional prognostic information beyond traditional risk factors in the ESKD population. Identifying high-risk patients through the CERT2 score could allow for more personalized management strategies, such as intensified monitoring or therapeutic interventions.

In addition to outperforming traditional biomarkers, the CERT2 risk score demonstrated superior predictive performance compared with widely used CV risk estimation tools, including SCORE2 and ASCVD risk scores, in the AURORA cohort. The CERT2 risk score showed stronger associations with study end points than SCORE2 and ASCVD. Furthermore, incorporating the CERT2 risk score into models that included either SCORE2 or ASCVD significantly improved the overall predictive performance, highlighting the incremental prognostic value of this lipid-based biomarker in patients with ESKD.

Strengths of the study include the two independent patient cohorts from the AURORA and 4D trials with large numbers of patients. Although the AURORA and 4D cohorts were enrolled over 2 decades ago, they remain among the most robust and comprehensively characterized hemodialysis populations. As both cohorts derived from randomized controlled trials, clinical patient data were collected according to strict protocols, and end points were centrally adjudicated. It is worth noting that all the patients enrolled in the 4D trial had type 2 diabetes mellitus, whereas in the AURORA trial, only about quarter of the patients had type 2 diabetes mellitus. Moreover, the AURORA cohort included 2311 patients, which is twice the number of patients enrolled in the 4D trial. Those differences do not allow for adequate comparison of the two study cohorts; therefore, we focus our approach on evaluating the utility of the CERT2 score within two distinct hemodialysis populations.

Both cohorts focus exclusively on European patients, which may limit the generalizability of our findings to more diverse populations. We also acknowledge the absence of dietary intake data as a limitation. Although diet may influence ceramide levels, plasma ceramides are also modulated by metabolic and inflammatory processes. Thus, they remain valid biomarkers in this clinical context.

As previously mentioned, both the AURORA and 4D cohorts were enrolled over 20 years ago, and as such the study populations do not fully reflect current dialysis practices. In both cohorts, <10% of the patients received hemodiafiltration, a modality that has gained widespread use in recent years and has been associated with lower mortality rates. The limited use of hemodiafiltration in these datasets may affect the generalizability of our results. In addition, the arteriovenous fistula was the predominant form of vascular access. This contrasts with current clinical trends where central venous catheters are more commonly used. As vascular access is an independent predictor of CV outcomes, these differences highlight the need to validate CERT2 in more contemporary dialysis populations.

In conclusion, the CERT2 risk score offers a novel lipid-based approach to CV risk stratification in patients with ESKD. On the basis of simple panel of plasma lipids, it effectively identifies individuals at an increased risk of major CV events and all-cause mortality, outperforming traditional biomarkers and more complex risk scores. Its prognostic utility applies to both diabetic and nondiabetic patients with ESKD on hemodialysis enhancing its broad clinical relevance. Integration of the CERT2 risk score into clinical practice may aid in identifying patients that might require more intensive preventive care because of elevated CV risk. Future studies should aim to validate CERT2 in the contemporary dialysis population and evaluate its utility in personalized interventions to reduce CV burden.

## Supplementary Material

SUPPLEMENTARY MATERIAL

## Data Availability

Original data generated for the study will be made available upon reasonable request to the corresponding author. Data Type: Aggregated Data. Reason for Restricted Access: The data analyzed in this study are available upon request from the corresponding author to ensure appropriate use and address ethical considerations regarding participant privacy.

## Declarative Statements

This study includes clinical experimentation and complies with the Declaration of Helsinki.

## Supplemental Material

Supplemental Table 1. Components and calculation methods for the CERT1 and CERT2 scores.

Supplemental Table 2. Description of CV risk scores (SCORE2, ASCVD) in AURORA population (*n*=2292).

Supplemental Table 3. Lipid species association with all-cause death and CV end points in the AURORA trial.

Supplemental Table 4. Lipid species association with all-cause death and CV end points in the 4D trial.

Supplemental Table 5. Predictive performance of CERT2 score in the AURORA trial: univariable c-index, comparison with LDL cholesterol, and added prognostic value.

Supplemental Table 6. Predictive performance of CERT2 score in the 4D trial: univariable c-index, comparison with LDL cholesterol, and added prognostic value.

Supplemental Table 7. Association of CV risk scores (SCORE2, ASCVD) with CV death, all-cause death, and three-point MACE in the AURORA trial.

Supplemental Figure 1. Flowchart of cohort selection for (A) AURORA and (B) 4D.
