# Supplementary material for: Predictive Performance of the Cardiovascular Event Risk Test 2 Risk Score in Hemodialysis Patients with ESKD
Source: Clin J Am Soc Nephrol. 2025 Nov 12;20(12):1683–95. doi: 10.2215/CJN.0000000831 (PMC12708377; doi:10.2215/CJN.0000000831)
Supplement: SUPPLEMENTARY MATERIAL [file cjasn-20-1683-s001.pdf]

ASN American Society of Nephrology
JASN Journal of the American Society of Nephrology
KIDNEY360 Accessing Our World From Every Angle
CJASN Clinical Journal of the American Society of Nephrology

ASN Journal Disclosure Form

As per ASN journal policy, I have disclosed any financial relationships or commitments I have held in the past 36 months as included below. I have listed my Current Employer below to indicate there is a relationship requiring disclosure. If no relationship exists, my Current Employer is not listed.

C. Drechsler reports the following:
Employer: University Hospital Wuerzburg

I understand that the information above will be published within the journal article, if accepted, and that failure to comply and/or to accurately and completely report the potential financial conflicts of interest could lead to the following: 1) Prior to publication, article rejection, or 2) Post-publication, sanctions ranging from, but not limited to, issuing a correction, reporting the inaccurate information to the authors' institution, banning authors from submitting work to ASN journals for varying lengths of time, and/or retraction of the published work.

Name: Christiane Drechsler
Manuscript ID: CJASN-2025-000445R2
Manuscript Title: Predictive Performance of the CERT2 Risk Score in Hemodialysis Patients with End-Stage Kidney Disease
Date of Completion: October 13, 2025
Disclosure Updated Date: October 13, 2025

ASN American Society of Nephrology · JASN Journal of the American Society of Nephrology · KIDNEY360 Accessing Our World From Every Angle · CJASN Clinical Journal of the American Society of Nephrology

ASN Journal Disclosure Form

As per ASN journal policy, I have disclosed any financial relationships or commitments I have held in the past 36 months as included below. I have listed my Current Employer below to indicate there is a relationship requiring disclosure. If no relationship exists, my Current Employer is not listed.

K. Duarte has nothing to disclose.

I understand that the information above will be published within the journal article, if accepted, and that failure to comply and/or to accurately and completely report the potential financial conflicts of interest could lead to the following: 1) Prior to publication, article rejection, or 2) Post-publication, sanctions ranging from, but not limited to, issuing a correction, reporting the inaccurate information to the authors' institution, banning authors from submitting work to ASN journals for varying lengths of time, and/or retraction of the published work.

Name: Kévin Duarte
Manuscript ID: CJASN-2025-000445R3
Manuscript Title: Predictive Performance of the CERT2 Risk Score in Hemodialysis Patients with End-Stage Kidney Disease
Date of Completion: September 2, 2025
Disclosure Updated Date: September 2, 2025

ASN American Society of Nephrology · JASN Journal of the American Society of Nephrology · KIDNEY360 Accessing Our World From Every Angle · CJASN Clinical Journal of the American Society of Nephrology

ASN Journal Disclosure Form

As per ASN journal policy, I have disclosed any financial relationships or commitments I have held in the past 36 months as included below. I have listed my Current Employer below to indicate there is a relationship requiring disclosure. If no relationship exists, my Current Employer is not listed.

N. Girerd reports the following:
Employer: Université de Lorraine and Nancy University Hospital, Nancy, France; Honoraria: AstraZeneca, Bayer, Boehringer, Cardiostory, Lilly, Echosens, NP medical, Novartis, Novo Nordisk, Roche diagnostics; and Advisory or Leadership Role: Former Board member of the Heart Failure association from the ESC.

I understand that the information above will be published within the journal article, if accepted, and that failure to comply and/or to accurately and completely report the potential financial conflicts of interest could lead to the following: 1) Prior to publication, article rejection, or 2) Post-publication, sanctions ranging from, but not limited to, issuing a correction, reporting the inaccurate information to the authors' institution, banning authors from submitting work to ASN journals for varying lengths of time, and/or retraction of the published work.

Name: Nicolas Girerd
Manuscript ID: CJASN-2025-000445R2
Manuscript Title: Predictive Performance of the CERT2 Risk Score in Hemodialysis Patients with End-Stage Kidney Disease
Date of Completion: July 18, 2025
Disclosure Updated Date: July 18, 2025

ASN American Society of Nephrology
JASN Journal of the American Society of Nephrology
KIDNEY360 Accessing Our World From Every Angle
CJASN Clinical Journal of the American Society of Nephrology

ASN Journal Disclosure Form

As per ASN journal policy, I have disclosed any financial relationships or commitments I have held in the past 36 months as included below. I have listed my Current Employer below to indicate there is a relationship requiring disclosure. If no relationship exists, my Current Employer is not listed.

M. Kleber reports the following:
Employer: SYNLAB Holding Deutschland GmbH

I understand that the information above will be published within the journal article, if accepted, and that failure to comply and/or to accurately and completely report the potential financial conflicts of interest could lead to the following: 1) Prior to publication, article rejection, or 2) Post-publication, sanctions ranging from, but not limited to, issuing a correction, reporting the inaccurate information to the authors' institution, banning authors from submitting work to ASN journals for varying lengths of time, and/or retraction of the published work.

Name: Marcus E. Kleber
Manuscript ID: CJASN-2025-000445R1
Manuscript Title: Predictive Performance of the CERT2 Risk Score in Hemodialysis Patients with End-Stage Kidney Disease
Date of Completion: July 1, 2025
Disclosure Updated Date: July 1, 2025

ASN American Society of Nephrology
JASN Journal of the American Society of Nephrology
KIDNEY360 Accessing Our World From Every Angle
CJASN Clinical Journal of the American Society of Nephrology

ASN Journal Disclosure Form

As per ASN journal policy, I have disclosed any financial relationships or commitments I have held in the past 36 months as included below. I have listed my Current Employer below to indicate there is a relationship requiring disclosure. If no relationship exists, my Current Employer is not listed.

R. Laaksonen reports the following:
Employer: Zora Biosciences Oy; Ownership Interest: Zora Biosciences Oy; Patents or Royalties: Zora Biosciences Oy; and Advisory or Leadership Role: Zora Biosciences Oy.

I understand that the information above will be published within the journal article, if accepted, and that failure to comply and/or to accurately and completely report the potential financial conflicts of interest could lead to the following: 1) Prior to publication, article rejection, or 2) Post-publication, sanctions ranging from, but not limited to, issuing a correction, reporting the inaccurate information to the authors' institution, banning authors from submitting work to ASN journals for varying lengths of time, and/or retraction of the published work.

Name: Reijo Laaksonen
Manuscript ID: CJASN-2025-000445R1
Manuscript Title: Predictive Performance of the CERT2 Risk Score in Hemodialysis Patients with End-Stage Kidney Disease
Date of Completion: June 30, 2025
Disclosure Updated Date: June 30, 2025

ASN American Society of Nephrology · JASN Journal of the American Society of Nephrology · KIDNEY360 Accessing Our World From Every Angle · CJASN Clinical Journal of the American Society of Nephrology

ASN Journal Disclosure Form

As per ASN journal policy, I have disclosed any financial relationships or commitments I have held in the past 36 months as included below. I have listed my Current Employer below to indicate there is a relationship requiring disclosure. If no relationship exists, my Current Employer is not listed.

W. März reports the following:
Employer: Synlab Holding Deutschland GmbH; Consultancy: AMGEN, Sanofi, Amryt Pharmaceuticals, Abbott Diagnostics, Akzea Therapeutics, Novartis, SOBI, Arrowhead, Ultragenyx, Boehringer Ingelheim; Research Funding: AMGEN, Sanofi, Abbott Diagnostics, Boehringer Ingelheim, Novartis, SOBI; Honoraria: AMGEN, Sanofi, Amryt Pharmaceuticals, Abbott Diagnostics, Akzea Therapeutics, Novartis, SOBI, Arrowhead, Ultragenyx, Boehringer Ingelheim; Patents or Royalties: LURIC Study GmbH; and Speakers Bureau: AMGEN, Sanofi, Amryt Pharmaceuticals, Abbott Diagnostics, Akzea Therapeutics, Novartis, SOBI, Arrowhead, Ultragenyx, Boehringer Ingelheim.

I understand that the information above will be published within the journal article, if accepted, and that failure to comply and/or to accurately and completely report the potential financial conflicts of interest could lead to the following: 1) Prior to publication, article rejection, or 2) Post-publication, sanctions ranging from, but not limited to, issuing a correction, reporting the inaccurate information to the authors' institution, banning authors from submitting work to ASN journals for varying lengths of time, and/or retraction of the published work.

Name: Winfried März
Manuscript ID: CJASN-2025-000445R3
Manuscript Title: Predictive Performance of the CERT2 Risk Score in Hemodialysis Patients with End-Stage Kidney Disease
Date of Completion: November 6, 2025
Disclosure Updated Date: October 22, 2025

ASN Journal Disclosure Form

As per ASN journal policy, I have disclosed any financial relationships or commitments I have held in the past 36 months as included below. I have listed my Current Employer below to indicate there is a relationship requiring disclosure. If no relationship exists, my Current Employer is not listed.

P. Rossignol reports the following:
Employer: University of Lorraine-Clinical Investigation Center-INSERM-CHRU of Nancy; and Princess Grace Hospital, Monaco; and Centre d'Hémodialyse Privé de Monaco; Consultancy: Dr. ROSSIGNOL reports personal fees from Alexion, Astra-Zeneca, Bayer, Boehringer Ingelheim, CinCor, Idorsia, KBP, NovoNordisk, Sanofi, Sequana medical, Servier, Vera Therapeutics, Vifor; Ownership Interest: G3P (stock options); Research Funding: Vifor Fresenius Medical Care Renal Pharma and Relypsa Inc., a Vifor Pharma Group Company; Honoraria: Dr. ROSSIGNOL reports personal fees from Alexion, Astra-Zeneca, Bayer, Boehringer Ingelheim, CinCor, Idorsia, KBP, NovoNordisk, Sanofi, Sequana medical, Servier, Vera Therapeutics, Vifor; Advisory or Leadership Role: KDIDO executive committee member since 2023; and Speakers Bureau: Dr. ROSSIGNOL reports personal fees from Alexion, Astra-Zeneca, Bayer, Boehringer Ingelheim, CinCor, Idorsia, KBP, NovoNordisk, Sanofi, Sequana medical, Servier, Vera Therapeutics, Vifor.

I understand that the information above will be published within the journal article, if accepted, and that failure to comply and/or to accurately and completely report the potential financial conflicts of interest could lead to the following: 1) Prior to publication, article rejection, or 2) Post-publication, sanctions ranging from, but not limited to, issuing a correction, reporting the inaccurate information to the authors' institution, banning authors from submitting work to ASN journals for varying lengths of time, and/or retraction of the published work.

Name: Patrick Rossignol
Manuscript ID: CJASN-2025-000445R3
Manuscript Title: Predictive Performance of the CERT2 Risk Score in Hemodialysis Patients with End-Stage Kidney Disease
Date of Completion: October 16, 2025
Disclosure Updated Date: October 16, 2025

ASN American Society of Nephrology
JASN Journal of the American Society of Nephrology
KIDNEY360 Accessing Our World From Every Angle
CJASN Clinical Journal of the American Society of Nephrology

ASN Journal Disclosure Form

As per ASN journal policy, I have disclosed any financial relationships or commitments I have held in the past 36 months as included below. I have listed my Current Employer below to indicate there is a relationship requiring disclosure. If no relationship exists, my Current Employer is not listed.

H. Scharnagl reports the following:
Research Funding: Abbott ; Amgen

I understand that the information above will be published within the journal article, if accepted, and that failure to comply and/or to accurately and completely report the potential financial conflicts of interest could lead to the following: 1) Prior to publication, article rejection, or 2) Post-publication, sanctions ranging from, but not limited to, issuing a correction, reporting the inaccurate information to the authors' institution, banning authors from submitting work to ASN journals for varying lengths of time, and/or retraction of the published work.

Name: Hubert Scharnagl
Manuscript ID: CJASN-2025-000445R2
Manuscript Title: Predictive performance of the CERT2 risk score in hemodialysis patients with end-stage kidney disease in
Date of Completion: July 14, 2025
Disclosure Updated Date: July 14, 2025

ASN
American Society of Nephrology
JASN
Journal of the American Society of Nephrology
KIDNEY360
Accessing Our World From Every Angle
CJASN
Clinical Journal of the American Society of Nephrology

ASN Journal Disclosure Form

As per ASN journal policy, I have disclosed any financial relationships or commitments I have held in the past 36 months as included below. I have listed my Current Employer below to indicate there is a relationship requiring disclosure. If no relationship exists, my Current Employer is not listed.

C. Wanner reports the following:
Employer: University Hospital; Consultancy: Alexion, AstraZeneca, Bayer, Boehringer-Ingelheim, GSK, Idorsia, MSD, NovoNordisk, CSL-Vifor, VeraTX; Research Funding: University of Oxford, Sanofi, AstraZeneca; Honoraria: Amgen, Amicus, Astellas, AstraZeneca, Bayer, Boehringer-Ingelheim, Chiesi, FMC, Eli-Lilly, GSK, Novartis, Sanofi, Stadapharm, Takeda, CSL-Vifor; and Other Interests or Relationships: European Renal Association.

I understand that the information above will be published within the journal article, if accepted, and that failure to comply and/or to accurately and completely report the potential financial conflicts of interest could lead to the following: 1) Prior to publication, article rejection, or 2) Post-publication, sanctions ranging from, but not limited to, issuing a correction, reporting the inaccurate information to the authors' institution, banning authors from submitting work to ASN journals for varying lengths of time, and/or retraction of the published work.

Name: Christoph Wanner
Manuscript ID: CJASN-2025-000445R2
Manuscript Title: Predictive Performance of the CERT2 Risk Score in Hemodialysis Patients with End-Stage Kidney Disease
Date of Completion: July 11, 2025
Disclosure Updated Date: July 11, 2025

ASN Journal Disclosure Form

As per ASN journal policy, I have disclosed any financial relationships or commitments I have held in the past 36 months as included below. I have listed my Current Employer below to indicate there is a relationship requiring disclosure. If no relationship exists, my Current Employer is not listed.

A. Witoslawska has nothing to disclose.

I understand that the information above will be published within the journal article, if accepted, and that failure to comply and/or to accurately and completely report the potential financial conflicts of interest could lead to the following: 1) Prior to publication, article rejection, or 2) Post-publication, sanctions ranging from, but not limited to, issuing a correction, reporting the inaccurate information to the authors' institution, banning authors from submitting work to ASN journals for varying lengths of time, and/or retraction of the published work.

Name: Angelika Zaneta Witoslawska
Manuscript ID: CJASN-2025-000445R1
Manuscript Title: Predictive Performance of the CERT2 Risk Score in Hemodialysis Patients with End-Stage Kidney Disease
Date of Completion: July 11, 2025
Disclosure Updated Date: July 11, 2025

ASN
American Society of Nephrology
JASN
Journal of the American Society of Nephrology
KIDNEY360
Accessing Our World From Every Angle
CJASN
Clinical Journal of the American Society of Nephrology

ASN Journal Disclosure Form

As per ASN journal policy, I have disclosed any financial relationships or commitments I have held in the past 36 months as included below. I have listed my Current Employer below to indicate there is a relationship requiring disclosure. If no relationship exists, my Current Employer is not listed.

F. Zannad reports the following:
Employer: Inserm, CHU & Université de Lorraine; Consultancy: Alnylam, Bayer, Biopeutics, Boehringer, Cellprothera, Cereno, Centrix, Corteria, CVRx, CVCT, Lilly, Lupin, Merck, NovoNordisk, Opalia Recordati, Owkin, Ribocure, Roche, Viatris.; Ownership Interest: Cereno, CVCT; Honoraria: Alnylam, Bayer, Biopeutics, Boehringer, Cellprothera, Cereno, Centrix, Corteria, CVRx, CVCT, Lilly, Lupin, Merck, NovoNordisk, Opalia Recordati, Owkin, Ribocure, Riche, Viatris.; Advisory or Leadership Role: Alnylam, Bayer, Biopeutics, Boehringer, Cellprothera, Cereno, Corteria, CVRx, Merck, Owkin, Ribocure, Roche; Speakers Bureau: Bayer, Boehringer, Centrix, CVRx, Lupin, Opalia Recordati, Merck, NovoNordisk, Viatris.; and Other Interests or Relationships: Polygon, Cereno pharmaceutical and CVCT.

I understand that the information above will be published within the journal article, if accepted, and that failure to comply and/or to accurately and completely report the potential financial conflicts of interest could lead to the following: 1) Prior to publication, article rejection, or 2) Post-publication, sanctions ranging from, but not limited to, issuing a correction, reporting the inaccurate information to the authors' institution, banning authors from submitting work to ASN journals for varying lengths of time, and/or retraction of the published work.

Name: Faiez Zannad
Manuscript ID: CJASN-2025-000445R3
Manuscript Title: Predictive Performance of the CERT2 Risk Score in Hemodialysis Patients with End-Stage Kidney Disease
Date of Completion: November 11, 2025
Disclosure Updated Date: November 11, 2025
