# Supplementary material for: Predictive Performance of the Cardiovascular Event Risk Test 2 Risk Score in Hemodialysis Patients with ESKD
Source: Clin J Am Soc Nephrol. 2025 Nov 12;20(12):1683–95. doi: 10.2215/CJN.0000000831 (PMC12708377; doi:10.2215/CJN.0000000831)

Supplemental Material

Table of Contents

Supplemental Table 1. Components and calculation methods for the CERT1 and CERT2 risk scores.

Supplemental Table 2. Description of CV risk scores (SCORE2, ASCVD) in AURORA population (n=2292).

Supplemental Table 3. Lipid species association with all-cause death and cardiovascular endpoints in the AURORA trial.

Supplemental Table 4. Lipid species association with all-cause death and cardiovascular endpoints in the 4D trial.

Supplemental Table 5. Predictive performance of CERT2 score in the AURORA trial: univariable c-index, comparison with LDL Cholesterol, and added prognostic value.

Supplemental Table 6. Predictive performance of CERT2 score in the 4D trial: univariable c-index, comparison with LDL Cholesterol, and added prognostic value.

Supplementary Table 7. Association of CV risk scores (SCORE2, ASCVD) with CV death, all-cause death, and 3-point MACE in the AURORA trial.

Supplemental Figure 1. Flowchart of cohort selection for (A) AURORA and (B) 4D.

Supplemental Table 1. Components and calculation methods for the CERT1 and CERT2 scores. (A) CERT1 is composed of three individual ceramide species, and three ceramide/ceramide ratios. CERT2 is composed of one ceramide/ceramide ratio, two ceramide/phosphatidylcholine ratios and one individual phosphatidylcholine species. For both CERT1 and CERT2 scores, individual lipid values were compared to the distribution of the entire study population. (B) Each lipid variable was categorized into quartiles (Q1–Q4), and a point score was assigned accordingly. (C) Based on the total CERT score (0-12), individuals were stratified into four risk categories. (D) The CERT2 risk score was calculated by summing the point values of each lipid marker.

Cer, ceramide; CERT2, cardiovascular event risk score; PC, phospholipid; Q, quartile.

A

**Score components**						
CERT1	Cer(d18:1/16:0)	Cer(d18:1/18:0)	Cer(d18:1/24:1)	Cer(d18:1/16:0)/ Cer(d18:1/24:0)	Cer(d18:1/18:0)/ Cer(d18:1/24:0)	Cer(d18:1/24:1)/ Cer(d18:1/24:0)
CERT2	Cer(d18:1/24:1)/ Cer(d18:1/24:0)	Cer(d18:1/16:0)/ PC 16:0/22:5	Cer(d18:1/18:0)/ PC 14:0/22:6	PC 16:0/16:0	-	-

B

**Quartiles**				
	Q1	Q2	Q3	Q4
CERT1	+0	+0	+1	+2
CERT2	+0	+1	+2	+3

C

**Risk group**				
	Low	Moderate	Increased	High
CERT1	0-2	3-6	7-9	10-12
CERT2	0-3	4-6	7-8	9-12

D

**CERT2**	**Q1**	**Q2**	**Q3**	**Q4**	**Score**
Cer(d18:1/24:1) / Cer(d18:1/24:0)	0	1	2	3	0-12
Cer(d18:1/16:0) / PC(16:0/22:5)	0	1	2	3	
Cer(d18:1/18:0) / PC(14:0/22:6)	0	1	2	3	
PC(16:0/16:0)	0	1	2	3	

Supplemental Table 2. Description of CV risk scores (SCORE2, ASCVD) in AURORA population (n=2292)**.**

	Mean ± SD / n (%)	Median (Q1 - Q3)
**SCORE2 – Very high-risk region**		
10-year CV risk (%)	24.0 ± 13.2	22.2 (12.9 - 33.9)
Category of 10-year CV risk		
Low risk	56 (2 %)	
Moderate risk	314 (14 %)	
High risk	1922 (84 %)	
**ASCVD score**		
10-year CV risk (%)	17.3 ± 14.1	14 (6 - 24)
Category of 10-year CV risk		
Very low risk	362 (16 %)	
Low risk	316 (14 %)	
Moderate risk	842 (37 %)	
High risk	772 (34 %)	

*To compute SCORE2, it is necessary to define the risk region level (low, moderate, high, or very high), which depends on the patient's country of residence. Due to missing country-of-residence data in the AURORA cohort, and given that all participants had ESKD – a population considered to be at high CV risk – SCORE2 was calculated using the 'very-high-risk-region' classification.

Supplemental Table 3. Lipid species association with all-cause death and cardiovascular endpoints in the AURORA trial.

**AURORA**	**Model 1 (n=2311*)**			**Model 2 (n=2311*)**			**Model 3 (n=2269*)**		
	**HR per 1SD (CI 95 %)**	**p-value**	**Adjusted p-value****	**HR per 1SD (CI 95 %)**	**p-value**	**Adjusted p-value****	**HR per 1SD (CI 95 %)**	**p-value**	**Adjusted p-value****
**CV death**									
Log - Cer(d18:1/16:0)	1.17 (1.08 - 1.27)	**<0.001**	**<0.001**	1.16 (1.07 - 1.27)	**<0.001**	**0.001**	1.13 (1.03 - 1.23)	**0.008**	**0.010**
Log - Cer(d18:1/18:0)	1.11 (1.02 - 1.20)	**0.02**	**0.02**	1.10 (1.01 - 1.20)	**0.02**	**0.03**	1.04 (0.95 - 1.14)	0.40	0.42
Log - Cer(d18:1/24:0)	0.86 (0.79 - 0.93)	**<0.001**	**<0.001**	0.85 (0.79 - 0.93)	**<0.001**	**<0.001**	0.88 (0.81 - 0.95)	**0.002**	**0.003**
Log - Cer(d18:1/24:1)	1.06 (0.98 - 1.15)	0.16	0.18	1.03 (0.94 - 1.12)	0.52	0.54	1.02 (0.94 - 1.11)	0.63	0.64
Log - PC(14:0/22:6)	0.74 (0.68 - 0.80)	**<0.001**	**<0.001**	0.71 (0.65 - 0.77)	**<0.001**	**<0.001**	0.75 (0.69 - 0.82)	**<0.001**	**<0.001**
Log - PC(16:0/16:0)	1.12 (1.03 - 1.22)	**0.006**	**0.008**	1.08 (0.99 - 1.18)	0.08	0.09	1.08 (0.98 - 1.17)	0.11	0.12
Log - PC(16:0/22:5)	0.80 (0.74 - 0.87)	**<0.001**	**<0.001**	0.81 (0.75 - 0.88)	**<0.001**	**<0.001**	0.85 (0.78 - 0.93)	**<0.001**	**<0.001**
Log - Cer(d18:1/24:1) / Cer(d18:1/24:0)	1.25 (1.15 - 1.36)	**<0.001**	**<0.001**	1.22 (1.12 - 1.32)	**<0.001**	**<0.001**	1.17 (1.08 - 1.28)	**<0.001**	**<0.001**
Log - Cer(d18:1/16:0) / PC(16:0/22:5)	1.37 (1.26 - 1.48)	**<0.001**	**<0.001**	1.33 (1.23 - 1.45)	**<0.001**	**<0.001**	1.28 (1.17 - 1.40)	**<0.001**	**<0.001**
Log - Cer(d18:1/18:0) / PC(14:0/22:6)	1.39 (1.28 - 1.51)	**<0.001**	**<0.001**	1.42 (1.31 - 1.54)	**<0.001**	**<0.001**	1.33 (1.21 - 1.45)	**<0.001**	**<0.001**
**All-cause death**									
Log - Cer(d18:1/16:0)	1.17 (1.10 - 1.24)	**<0.001**	**<0.001**	1.16 (1.08 - 1.23)	**<0.001**	**<0.001**	1.09 (1.02 - 1.17)	**0.009**	**0.01**
Log - Cer(d18:1/18:0)	1.11 (1.05 - 1.18)	**<0.001**	**<0.001**	1.11 (1.04 - 1.18)	**0.001**	**0.002**	1.03 (0.96 - 1.10)	0.44	0.47
Log - Cer(d18:1/24:0)	0.83 (0.78 - 0.88)	**<0.001**	**<0.001**	0.83 (0.78 - 0.88)	**<0.001**	**<0.001**	0.85 (0.80 - 0.90)	**<0.001**	**<0.001**
Log - Cer(d18:1/24:1)	1.05 (0.99 - 1.12)	0.10	0.12	1.02 (0.95 - 1.08)	0.63	0.64	0.99 (0.93 - 1.06)	0.82	0.82
Log - PC(14:0/22:6)	0.78 (0.73 - 0.83)	**<0.001**	**<0.001**	0.75 (0.70 - 0.79)	**<0.001**	**<0.001**	0.80 (0.75 - 0.86)	**<0.001**	**<0.001**
Log - PC(16:0/16:0)	1.17 (1.10 - 1.24)	**<0.001**	**<0.001**	1.12 (1.05 - 1.19)	**<0.001**	**<0.001**	1.10 (1.03 - 1.17)	**0.004**	**0.006**
Log - PC(16:0/22:5)	0.82 (0.77 - 0.87)	**<0.001**	**<0.001**	0.83 (0.78 - 0.88)	**<0.001**	**<0.001**	0.88 (0.83 - 0.94)	**<0.001**	**<0.001**
Log - Cer(d18:1/24:1) / Cer(d18:1/24:0)	1.29 (1.21 - 1.37)	**<0.001**	**<0.001**	1.25 (1.17 - 1.33)	**<0.001**	**<0.001**	1.19 (1.12 - 1.27)	**<0.001**	**<0.001**
Log - Cer(d18:1/16:0) / PC(16:0/22:5)	1.34 (1.26 - 1.42)	**<0.001**	**<0.001**	1.30 (1.23 - 1.39)	**<0.001**	**<0.001**	1.20 (1.12 - 1.29)	**<0.001**	**<0.001**
Log - Cer(d18:1/18:0) / PC(14:0/22:6)	1.33 (1.26 - 1.42)	**<0.001**	**<0.001**	1.36 (1.28 - 1.45)	**<0.001**	**<0.001**	1.24 (1.16 - 1.32)	**<0.001**	**<0.001**
**3-point MACE**									
Log - Cer(d18:1/16:0)	1.17 (1.09 - 1.26)	**<0.001**	**<0.001**	1.17 (1.09 - 1.27)	**<0.001**	**0.001**	1.13 (1.05 - 1.23)	**0.002**	**0.003**
Log - Cer(d18:1/18:0)	1.12 (1.04 - 1.20)	**0.003**	**0.005**	1.12 (1.04 - 1.21)	**0.003**	**0.004**	1.06 (0.98 - 1.15)	0.18	0.19
Log - Cer(d18:1/24:0)	0.88 (0.82 - 0.94)	**<0.001**	**<0.001**	0.88 (0.82 - 0.95)	**<0.001**	**0.001**	0.90 (0.84 - 0.97)	**0.007**	**0.009**
Log - Cer(d18:1/24:1)	1.10 (1.02 - 1.18)	**0.01**	**0.02**	1.08 (1.00 - 1.16)	0.06	0.07	1.07 (0.99 - 1.15)	0.11	0.12
Log - PC(14:0/22:6)	0.78 (0.73 - 0.84)	**<0.001**	**<0.001**	0.76 (0.71 - 0.82)	**<0.001**	**<0.001**	0.80 (0.74 - 0.87)	**<0.001**	**<0.001**
Log - PC(16:0/16:0)	1.10 (1.02 - 1.19)	**0.01**	**0.01**	1.07 (0.99 - 1.15)	0.10	0.12	1.06 (0.98 - 1.14)	0.17	0.19

AURORA	Model 1 (n=2311*)			Model 2 (n=2311*)			Model 3 (n=2269*)		
	HR per 1SD (CI 95 %)	p-value	Adjusted p-value**	HR per 1SD (CI 95 %)	p-value	Adjusted p-value**	HR per 1SD (CI 95 %)	p-value	Adjusted p-value**
Log - PC(16:0/22:5)	0.83 (0.77 - 0.89)	**<0.001**	**<0.001**	0.84 (0.78 - 0.91)	**<0.001**	**<0.001**	0.89 (0.82 - 0.96)	**0.003**	**0.005**
Log - Cer(d18:1/24:1) / Cer(d18:1/24:0)	1.27 (1.18 - 1.37)	**<0.001**	**<0.001**	1.24 (1.14 - 1.33)	**<0.001**	**<0.001**	1.19 (1.10 - 1.29)	**<0.001**	**<0.001**
Log - Cer(d18:1/16:0) / PC(16:0/22:5)	1.32 (1.22 - 1.42)	**<0.001**	**<0.001**	1.29 (1.20 - 1.39)	**<0.001**	**<0.001**	1.22 (1.13 - 1.33)	**<0.001**	**<0.001**
Log - Cer(d18:1/18:0) / PC(14:0/22:6)	1.33 (1.23 - 1.43)	**<0.001**	**<0.001**	1.35 (1.25 - 1.45)	**<0.001**	**<0.001**	1.26 (1.16 - 1.36)	**<0.001**	**<0.001**

HR: hazard ratio; SD: standard deviation.

Model 1: unadjusted.

Model 2: adjusted for age, gender.

Model 3: adjusted for age, gender, body mass index, systemic hypertension, T2DM, smoking, log hsCRP, treatment group (rosuvastatin vs placebo).

*Models including Log - Cer(d18:1/24:0) had one fewer observation (i.e., n = 2310 for models 1/2, n = 2268 for model 3), those including Log - Cer(d18:1/24:1) had three fewer observations (i.e., n = 2308 for models 1/2, n = 2266 for model 3), and those including Log - Cer(d18:1/24:1) / Cer(d18:1/24:0) had four fewer observations (i.e., n = 2307 for models 1/2, n = 2265 for model 3).

**P-values adjusted for multiple comparisons using the Benjamini-Hochberg method across all 90 tests (10 lipid variables x 3 outcomes x 3 models).

Supplemental Table 4. Lipid species association with all-cause death and cardiovascular endpoints in the 4D trial.

**4D**	**Model 1 (n=1137)**			**Model 2 (n=1137)**			**Model 3 (n=1117)**		
	**HR per 1SD (CI 95 %)**	**p-value**	**Adjusted p-value****	**HR per 1SD (CI 95 %)**	**p-value**	**Adjusted p-value****	**HR per 1SD (CI 95 %)**	**p-value**	**Adjusted p-value****
**CV death**									
Log - Cer(d18:1/16:0)	1.16 (1.02 - 1.32)	**0.02**	0.05	1.15 (1.01 - 1.31)	**0.03**	0.07	1.08 (0.95 - 1.23)	0.26	0.34
Log - Cer(d18:1/18:0)	1.13 (0.99 - 1.28)	0.07	0.12	1.14 (1.00 - 1.30)	0.05	0.10	1.07 (0.93 - 1.23)	0.33	0.40
Log - Cer(d18:1/24:0)	0.94 (0.83 - 1.06)	0.32	0.39	0.94 (0.83 - 1.06)	0.31	0.39	0.93 (0.82 - 1.06)	0.28	0.37
Log - Cer(d18:1/24:1)	1.04 (0.91 - 1.17)	0.59	0.64	1.03 (0.91 - 1.17)	0.65	0.69	1.00 (0.88 - 1.13)	0.95	0.95
Log - PC(14:0/22:6)	0.90 (0.80 - 1.02)	0.10	0.15	0.88 (0.77 - 1.00)	0.05	0.10	0.90 (0.79 - 1.03)	0.12	0.19
Log - PC(16:0/16:0)	0.99 (0.90 - 1.10)	0.91	0.92	0.99 (0.89 - 1.10)	0.85	0.86	0.96 (0.85 - 1.09)	0.54	0.60
Log - PC(16:0/22:5)	0.91 (0.81 - 1.03)	0.14	0.21	0.92 (0.81 - 1.05)	0.21	0.31	0.94 (0.83 - 1.06)	0.31	0.39
Log - Cer(d18:1/24:1) / Cer(d18:1/24:0)	1.13 (0.99 - 1.27)	0.06	0.11	1.12 (0.99 - 1.27)	0.08	0.13	1.08 (0.95 - 1.23)	0.22	0.31
Log - Cer(d18:1/16:0) / PC(16:0/22:5)	1.25 (1.11 - 1.41)	**<0.001**	**0.002**	1.23 (1.08 - 1.39)	**0.001**	**0.006**	1.15 (1.01 - 1.30)	**0.04**	0.08
Log - Cer(d18:1/18:0) / PC(14:0/22:6)	1.19 (1.05 - 1.34)	**0.006**	**0.02**	1.21 (1.07 - 1.38)	**0.003**	**0.01**	1.14 (1.00 - 1.30)	**0.05**	0.09
**All-cause death**									
Log - Cer(d18:1/16:0)	1.27 (1.16 - 1.38)	**<0.001**	**<0.001**	1.25 (1.15 - 1.36)	**<0.001**	**<0.001**	1.15 (1.06 - 1.26)	**0.001**	**0.006**
Log - Cer(d18:1/18:0)	1.20 (1.10 - 1.31)	**<0.001**	**<0.001**	1.22 (1.12 - 1.33)	**<0.001**	**<0.001**	1.11 (1.01 - 1.21)	**0.03**	0.07
Log - Cer(d18:1/24:0)	0.97 (0.90 - 1.06)	0.51	0.59	0.97 (0.89 - 1.06)	0.50	0.58	0.95 (0.87 - 1.04)	0.25	0.34
Log - Cer(d18:1/24:1)	1.11 (1.02 - 1.21)	**0.01**	**0.04**	1.10 (1.01 - 1.20)	**0.02**	0.05	1.05 (0.96 - 1.14)	0.30	0.38
Log - PC(14:0/22:6)	0.92 (0.84 - 0.99)	**0.04**	0.08	0.89 (0.81 - 0.97)	**0.009**	**0.03**	0.90 (0.83 - 0.99)	**0.03**	0.06
Log - PC(16:0/16:0)	1.02 (0.96 - 1.07)	0.59	0.64	1.01 (0.96 - 1.07)	0.73	0.76	0.98 (0.92 - 1.05)	0.64	0.69
Log - PC(16:0/22:5)	0.91 (0.84 - 0.98)	**0.02**	0.05	0.92 (0.85 - 1.00)	0.06	0.12	0.95 (0.87 - 1.04)	0.25	0.34
Log - Cer(d18:1/24:1) / Cer(d18:1/24:0)	1.18 (1.08 - 1.28)	**<0.001**	**<0.001**	1.17 (1.07 - 1.27)	**<0.001**	**0.001**	1.12 (1.03 - 1.23)	**0.007**	**0.02**
Log - Cer(d18:1/16:0) / PC(16:0/22:5)	1.35 (1.25 - 1.47)	**<0.001**	**<0.001**	1.32 (1.22 - 1.43)	**<0.001**	**<0.001**	1.20 (1.10 - 1.31)	**<0.001**	**<0.001**
Log - Cer(d18:1/18:0) / PC(14:0/22:6)	1.21 (1.12 - 1.32)	**<0.001**	**<0.001**	1.25 (1.15 - 1.36)	**<0.001**	**<0.001**	1.16 (1.06 - 1.26)	**<0.001**	**0.004**

**4D**	**Model 1 (n=1137)**			**Model 2 (n=1137)**			**Model 3 (n=1117)**		
	**HR per 1SD (CI 95 %)**	**p-value**	**Adjusted p-value****	**HR per 1SD (CI 95 %)**	**p-value**	**Adjusted p-value****	**HR per 1SD (CI 95 %)**	**p-value**	**Adjusted p-value****
**3-point MACE**									
Log - Cer(d18:1/16:0)	1.14 (1.03 - 1.26)	**0.01**	**0.03**	1.12 (1.01 - 1.24)	**0.03**	0.07	1.08 (0.97 - 1.19)	0.16	0.24
Log - Cer(d18:1/18:0)	1.15 (1.04 - 1.28)	**0.006**	**0.02**	1.15 (1.04 - 1.27)	**0.009**	**0.03**	1.10 (0.99 - 1.23)	0.08	0.14
Log - Cer(d18:1/24:0)	0.94 (0.85 - 1.04)	0.22	0.32	0.94 (0.85 - 1.04)	0.21	0.31	0.94 (0.85 - 1.04)	0.22	0.32
Log - Cer(d18:1/24:1)	1.04 (0.94 - 1.15)	0.41	0.49	1.03 (0.94 - 1.14)	0.51	0.59	1.01 (0.91 - 1.12)	0.81	0.84
Log - PC(14:0/22:6)	0.88 (0.80 - 0.97)	**0.01**	**0.03**	0.83 (0.75 - 0.92)	**<0.001**	**0.003**	0.84 (0.76 - 0.94)	**0.002**	**0.007**
Log - PC(16:0/16:0)	0.98 (0.89 - 1.07)	0.62	0.67	0.97 (0.88 - 1.06)	0.49	0.58	0.94 (0.83 - 1.05)	0.26	0.35
Log - PC(16:0/22:5)	0.90 (0.82 - 1.00)	**0.04**	0.09	0.90 (0.82 - 1.00)	**0.05**	0.10	0.91 (0.83 - 1.01)	0.09	0.14
Log - Cer(d18:1/24:1) / Cer(d18:1/24:0)	1.13 (1.03 - 1.25)	**0.01**	**0.04**	1.12 (1.02 - 1.24)	**0.02**	0.05	1.10 (0.99 - 1.21)	0.08	0.13
Log - Cer(d18:1/16:0) / PC(16:0/22:5)	1.24 (1.12 - 1.36)	**<0.001**	**<0.001**	1.22 (1.10 - 1.34)	**<0.001**	**<0.001**	1.17 (1.06 - 1.30)	**0.003**	**0.01**
Log - Cer(d18:1/18:0) / PC(14:0/22:6)	1.22 (1.11 - 1.35)	**<0.001**	**<0.001**	1.27 (1.15 - 1.40)	**<0.001**	**<0.001**	1.22 (1.10 - 1.35)	**<0.001**	**<0.001**

HR: hazard ratio; SD: standard deviation

Model 1: unadjusted.

Model 2: adjusted for age, gender

Model 3: adjusted for age, gender, body mass index, systemic hypertension, T2DM, smoking, log hsCRP, treatment group (atorvastatin vs placebo).

**P-values adjusted for multiple comparisons using the Benjamini-Hochberg method across all 90 tests (10 lipid variables x 3 outcomes x 3 models).

Supplemental Table 5. Predictive performance of CERT2 score in the AURORA trial: univariable c-index, comparison with LDL Cholesterol, and added prognostic value.

AURORA	Endpoint	C-index (CI 95%)		Δ c-index (CI 95%)	p-value
		Model 1	Model 2		
Model 1: null model<br>Model 2: CERT2 score<br>n=2307	CV death	-	0.59 (0.57 - 0.62)	-	**<0.001**
	All-cause death	-	0.60 (0.58 - 0.61)	-	**<0.001**
	MACE	-	0.58 (0.56 - 0.60)	-	**<0.001**
Model 1: LDL-cholesterol<br>Model 2: CERT2 score<br>n=2294	CV death	0.53 (0.51 - 0.56)	0.59 (0.57 - 0.62)	0.06 (0.03 to 0.10)	**<0.001**
	All-cause death	0.54 (0.52 - 0.56)	0.60 (0.58 - 0.61)	0.06 (0.03 to 0.08)	**<0.001**
	MACE	0.52 (0.50 - 0.55)	0.58 (0.56 - 0.60)	0.06 (0.03 to 0.09)	**<0.001**
Model 1: LDL-cholesterol<br>Model 2: LDL-cholesterol + CERT2 score<br>n=2294	CV death	0.53 (0.51 - 0.56)	0.60 (0.58 - 0.63)	0.07 (0.04 to 0.10)	**<0.001**
	All-cause death	0.54 (0.52 - 0.56)	0.61 (0.59 - 0.63)	0.07 (0.05 to 0.09)	**<0.001**
	MACE	0.52 (0.50 - 0.55)	0.59 (0.57 - 0.61)	0.07 (0.04 to 0.09)	**<0.001**
Model 1: clinical model*<br>Model 2: clinical model* + CERT2 score<br>n=2265	CV death	0.64 (0.62 - 0.67)	0.66 (0.64 - 0.68)	0.02 (0.01 to 0.03)	**<0.001**
	All-cause death	0.65 (0.63 - 0.67)	0.66 (0.65 - 0.68)	0.02 (0.01 to 0.02)	**<0.001**
	MACE	0.64 (0.62 - 0.66)	0.65 (0.63 - 0.67)	0.01 (0.01 to 0.02)	**0.002**
Model 1: clinical model* + LDL-cholesterol<br>Model 2: clinical model* + LDL-cholesterol + CERT2 score<br>n=2264	CV death	0.65 (0.63 - 0.67)	0.67 (0.64 - 0.69)	0.02 (0.01 to 0.03)	**<0.001**
	All-cause death	0.66 (0.64 - 0.67)	0.67 (0.66 - 0.69)	0.02 (0.01 to 0.02)	**<0.001**
	MACE	0.64 (0.62 - 0.66)	0.65 (0.63 - 0.67)	0.01 (0.01 to 0.02)	**0.002**

*Clinical model included age, gender, body mass index, systemic hypertension, T2DM, smoking, log hsCRP and treatment group (atorvastatin vs placebo).

Supplemental Table 6. Predictive performance of CERT2 score in the 4D trial: univariable c-index, comparison with LDL Cholesterol, and added prognostic value.

**4D**	**Endpoint**	**C-index (CI 95%)**		**Δ c-index (CI 95%)**	**p-value**
		**Model 1**	**Model 2**		
Model 1: null model<br>Model 2: CERT2 score<br>(univariable c-index)<br>n=1137	CV death	-	0.56 (0.52 - 0.60)	-	**0.002**
	All-cause death	-	0.58 (0.56 - 0.61)	-	**<0.001**
	MACE	-	0.56 (0.53 - 0.59)	-	**<0.001**
Model 1: LDL-cholesterol<br>Model 2: CERT2 score<br>n=1060	CV death	0.52 (0.48 - 0.56)	0.56 (0.52 - 0.60)	0.04 (-0.01 to 0.10)	0.14
	All-cause death	0.50 (0.48 - 0.53)	0.58 (0.56 - 0.61)	0.07 (0.03 to 0.11)	**<0.001**
	MACE	0.50 (0.47 - 0.53)	0.56 (0.53 - 0.59)	0.06 (0.02 to 0.11)	**0.009**
Model 1: LDL-cholesterol<br>Model 2: LDL-cholesterol + CERT2 score<br>n=1060	CV death	0.52 (0.48 - 0.56)	0.57 (0.53 - 0.61)	0.05 (0.01 to 0.10)	**0.03**
	All-cause death	0.51 (0.48 - 0.53)	0.58 (0.56 - 0.61)	0.08 (0.04 to 0.11)	**<0.001**
	MACE	0.50 (0.47 - 0.53)	0.56 (0.53 - 0.59)	0.06 (0.02 to 0.10)	**0.004**
Model 1: clinical model*<br>Model 2: clinical model* + CERT2 score<br>n=1117	CV death	0.61 (0.58 - 0.65)	0.62 (0.58 - 0.66)	0.01 (-0.01 to 0.02)	0.39
	All-cause death	0.63 (0.61 - 0.66)	0.65 (0.62 - 0.67)	0.02 (0.00 to 0.03)	**0.008**
	MACE	0.59 (0.56 - 0.62)	0.60 (0.57 - 0.63)	0.01 (-0.01 to 0.03)	0.18
Model 1: clinical model* + LDL-cholesterol<br>Model 2: clinical model* + LDL-cholesterol + CERT2 score<br>n=1041	CV death	0.61 (0.57 - 0.64)	0.62 (0.58 - 0.65)	0.01 (-0.01 to 0.03)	0.30
	All-cause death	0.62 (0.60 - 0.65)	0.64 (0.61 - 0.66)	0.02 (0.00 to 0.03)	**0.01**
	MACE	0.58 (0.55 - 0.61)	0.60 (0.57 - 0.63)	0.01 (-0.01 to 0.03)	0.16

*Clinical model included age, gender, body mass index, systemic hypertension, T2DM, smoking, log hsCRP and treatment group (atorvastatin vs placebo).

Supplementary Table 7: Association of CV risk scores (SCORE2, ASCVD) with CV death, all-cause death, and 3-point MACE in the AURORA trial.

Nevents/N (%); Number of events/Total number of subjects in group

**AURORA**			**Nevents/N (%)**	**Unadjusted (n=2292)**	
				**HR (CI 95 %)**	**p-value**
**CV death**	SCORE2 – Very high-risk region				
		Low risk	9/56 (16.1%)	ref	-
		Moderate risk	43/314 (13.7%)	0.81 (0.39 - 1.66)	0.56
		High risk	532/1922 (27.7%)	1.94 (1.00 - 3.75)	**0.05**
**Death**	SCORE2 – Very high-risk region				
		Low risk	17/56 (30.4%)	ref	-
		Moderate risk	82/314 (26.1%)	0.81 (0.48 - 1.37)	0.43
		High risk	973/1922 (50.6%)	1.89 (1.17 - 3.05)	**0.009**
**MACE**	SCORE2 – Very high-risk region				
		Low risk	11/56 (19.6%)	ref	-
		Moderate risk	53/314 (16.9%)	0.82 (0.43 - 1.56)	0.54
		High risk	652/1922 (33.9%)	2.00 (1.10 - 3.62)	**0.02**
**CV death**	ASCVD score				
		Very low risk	69/362 (19.1%)	ref	-
		Low risk	55/316 (17.4%)	0.91 (0.64 - 1.30)	0.61
		Moderate risk	217/842 (25.8%)	1.48 (1.13 - 1.94)	**0.005**
		High risk	243/772 (31.5%)	1.94 (1.49 - 2.54)	**<0.001**
**Death**	ASCVD score				
		Very low risk	123/362 (34.0%)	ref	-
		Low risk	103/316 (32.6%)	0.96 (0.74 - 1.24)	0.73
		Moderate risk	393/842 (46.7%)	1.51 (1.24 - 1.85)	**<0.001**
		High risk	453/772 (58.7%)	2.06 (1.69 - 2.51)	**<0.001**
**MACE**	ASCVD score				
		Very low risk	79/362 (21.8%)	ref	-
		Low risk	80/316 (25.3%)	1.19 (0.88 - 1.63)	0.26
		Moderate risk	264/842 (31.4%)	1.60 (1.25 - 2.06)	**<0.001**
		High risk	293/772 (38.0%)	2.10 (1.64 - 2.69)	**<0.001**

Supplemental Figure 1. Flowchart of cohort selection for (A) AURORA and (B) 4D.

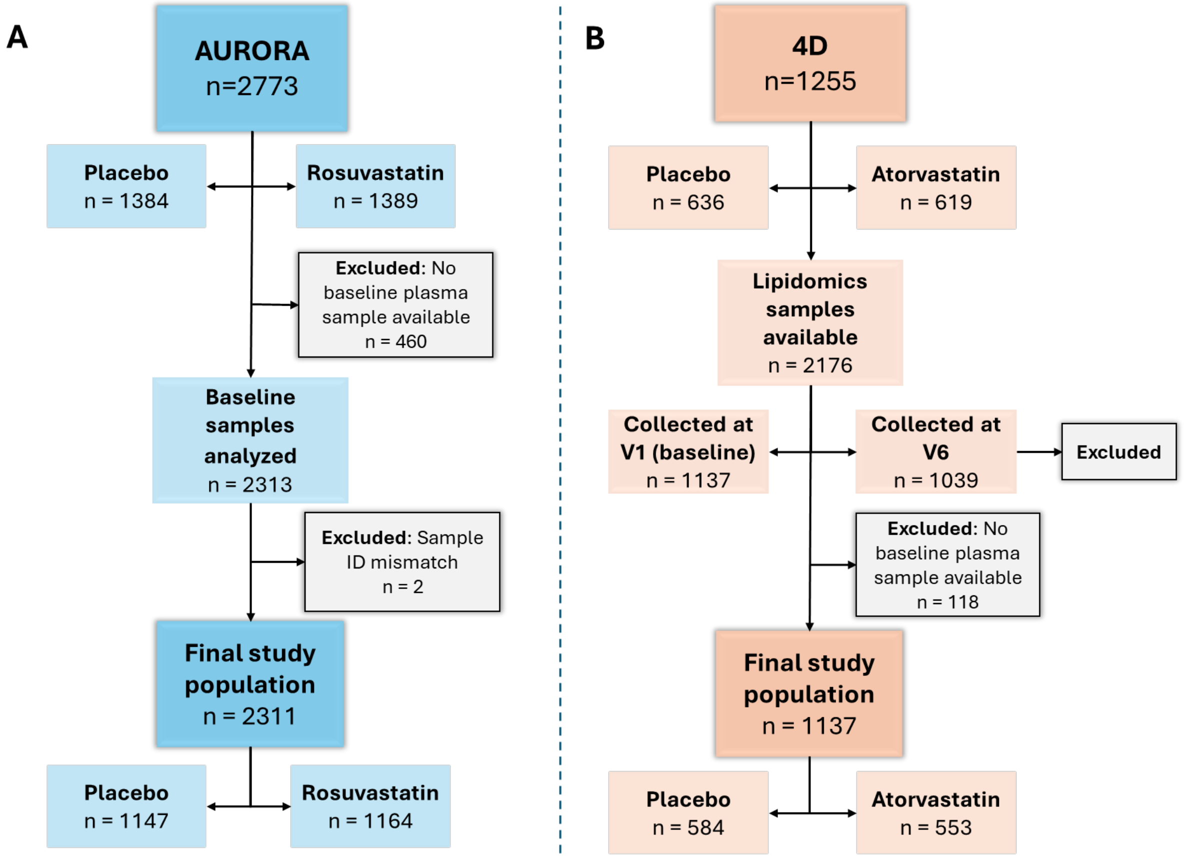

Supplement: SUPPLEMENTARY MATERIAL [file cjasn-20-1683-s002.pdf]
